# Effects of Coated Sodium Butyrate and Polysaccharides From *Cordyceps cicadae* on Intestinal Tissue Morphology and Ileal Microbiome of Squabs

**DOI:** 10.3389/fvets.2022.813800

**Published:** 2022-03-04

**Authors:** Hanxue Sun, Yali Liu, Tao Zeng, Guoqin Li, Zhengrong Tao, Xueqin Zhou, Jihui Wen, Xiaoyan Chen, Wenwu Xu, Lizhi Lu, Hongguo Cao

**Affiliations:** ^1^College of Animal Science and Technology, Anhui Agricultural University, Hefei, China; ^2^Animal Husbandry and Veterinary Institute, Zhejiang Academy of Agricultural Sciences, Hangzhou, China; ^3^Zhejiang Animal Husbandary Promotion Station, Hangzhou, China; ^4^Huzhou Huajia Special Breeding Co.Ltd, Huzhou, China; ^5^Aofeng Pigeon Industry in Pingyang County, Wenzhou, China

**Keywords:** coated sodium butyrate, polysaccharides from *Cordyceps cicadae*, squab, intestinal morphology, intestinal flora

## Abstract

This experiment was conducted to investigate the effects of dietary supplementation with different levels of coated sodium butyrate (CSB) and polysaccharides extracted from *Cordyceps cicadae* (CCP) on growth performance, intestinal tissue morphology and ileum microbiome in squabs. A total of 420 1-day-old squabs were randomly divided into seven groups with 5 replicates each and 12 squabs per replicate. The squabs were fed basal diet (control group) and basal diet supplemented with different levels of CSB (275, 550, and 1,100 mg/kg, groups CSB-275, CSB-550, CSB-1100) and CCP (27.5, 55, and 110 mg/kg, groups CCP-27.5, CCP-55, and CCP-110), respectively. The experiment was conducted for 28 days. The results revealed that the final BW and average daily gain concentration were higher (*P* < 0.05) in squabs of CSB-275 and CCP-110 groups than those in the CON group. Comparing with control group, the squabs in the groups CSB-275, CSB-550, and CCP-55 obtained higher villus height/crypt depth (VH/CD) of the duodenum and higher VH of the jejunum (*P* < 0.05). Operational taxonomic units in the groups CSB-550 and CCP-27.5 were also increased (*P* < 0.05). Regarding the relative abundance of flora, the *Actinobacteria* abundance in the groups CSB-550, CSB-1100, and CCP-55 were higher than in control group (*P* < 0.05), and the *Aeriscardovia* abundance of CSB-275, CSB-550, CSB-1100, and CCP-110 were elevated (*P* < 0.05). However, the *Enterococcus* abundance in CSB-275, CSB-550, CSB-1100, and CCP-27.5 decreased (*P* < 0.05). In summary, results obtained in the present study indicate that CSB and CCP can improve growth performance, intestinal microbial balance and gut health of squabs.

## Introduction

With the continuous improvement in living standards, consumer have higher expectations regarding the nutritional content and taste of meat. Due to the low cholesterol and high protein content, pigeon meat can be used as a valuable inclusive component of the human diet (1). In animal production, antibiotics are used traditionally with an aim to improve the growth performance and prevent the diseases. However, overdose use of antibiotics in animal diets gave rise to antimicrobial resistance (2), this problem has been defined as one of the greatest threats to public health in medicine in the twenty-first century (3). Butyric acid is a short-chain acid. Butyric acid is produced by microbial fermentation in the intestine (4) and is a fast energy source for intestinal epithelial cells (5). Sodium butyrate (SB) is a common feed additive. Previous studies have shown that dietary sodium butyrate supplementation can improve animal immune responses (5) and growth performance (6, 7). However, birds are reluctant to eat SB due to its unappetizing smell and therefore its digestibility decreases (8). To resolve this problem, SB is coated with a fat layer to form coated sodium butyrate (CSB), which not only improves the smell but also allows sodium butyrate slow release in the digestive tract (9). The *cicada* flower is a traditional Chinese medicine. According to *Pharmacopeia, Cordyceps cicada* flowers are rich in polysaccharides, adenosine, and other substances, which were proven to be functional ingredients for protecting the liver (10). Polysaccharides from *Cordyceps cicadae* (CCP) enhanced renal function (11), and several studies reported beneficial effects of CCP on treating hyperlipidemia, hyperglycemia, and liver injury (12, 13). Moreover, it could enhance the immune capacity of the body (14, 15).

Microbes can help decomposition of cellulose into volatile fatty acids, and regulate the intestinal environment to promote nutrient absorption (16). In addition, the normal intestinal flora can promote the development and maturation of the host immune system and maintain the health of the host (17, 18). Guilloteau et al. give an overview of the favorable effects of butyrate on the gastrointestinal tract (GIT) of broilers, including stimulation of growth performance, anti-inflammatory effects, maintenance of intestinal epithelial barrier integrity, and reduction of Salmonella colonization (19, 20), CSB and CCP have similar effects on improving animal immune ability (14, 15, 21). However, there is very limited information on effects of CSB and CCP on intestinal microbial population and morphology in squabs. The objective of the present research was to evaluate the use of CSB and CCP in meat pigeon industry its effect on intestinal morphology and microflora of squabs and determine the optimal supplemental amount, so as to provide theoretical basis for the future production of meat pigeons.

## Materials and Methods

### Experimental Design

A total of 420 1-day-old squabs were randomly divided into seven groups with 5 replicates each and 12 squabs per replicate. The squabs in control group were fed a basal diet; the squabs in groups CSB-275, CSB-550, and CSB-1100 were fed the basal diet supplemented with 275, 550, and 1,100 mg/kg CSB, respectively, and the squabs in groups CCP-27.5, CCP-55, and CCP-110 were fed the basal diet supplemented with 27.5, 55, and 110 mg/kg CCP, respectively. The experiment lasted 28 days.

### Experimental Materials

The CCP was provided by the Zhejiang Institute of Subtropical Crops. *Cordyceps cicadae* was dried at 105°C for 2 h, and then turned to 65°C for drying. The mycelium was crushed into 150 mesh *Cordyceps cicadae* powder with a pulverizer. Pure water was added according to the ratio of liquid to material 10:1. The extract obtained by direct water extraction (60°C water bath for 1 h) was evaporated and concentrated under reduced pressure at 60°C, and then added with 95% ethanol of 4 times its volume. The crude polysaccharide was precipitated in a refrigerator at 4°C overnight, The solvent was evaporated in an oven at 60°C to obtain the crude polysaccharide of cicada flower. The final CCP used for the experiment was obtained using the Sevag method and macroporous resin method. The content of polysaccharide measured by spectrophotometry was 24.26 mg/g. CSB was purchased from Hangzhou Technology Co. Ltd, and white king pigeons were provided by Aofeng Pigeon Industry Co. Ltd, Pingyang County, Wenzhou City, Zhejiang Province, China.

### Experimental Rations and Feeding Management

According to the Technical Standard for Breeding and Management of American King Pigeon Breeds (DB34/T541-2005), adult breeding pigeons were fed with a basal diet (grain raw food), and the composition and nutrient level of the basal diet were shown in Table 1. The traditional “2 + 2” feeding mode was adopted, each pair of adult breeding pigeons feed two squabs. The breeding pigeons were free to eat, drink, and access healthy sand daily during 14 h of light. The roost was cleaned regularly, and pigeons were observed to record their mental condition, illnesses, and deaths. The procedures were performed in accordance with the guidelines issued by the European Commission, and every effort was made to maximize humane treatment of the animals examined. All animals were euthanized by pentobarbital sodium for tissue collection.

**Table 1:** Composition and nutrient levels of the basal diet (as-dry basis) %.

**Ingredients**	**Content**	**Nutrient levels^b^**	**Content**
Corn	50.0	ME/(MJ/kg)	13.67
Pea	27.0	CP	13.83
Wheat	10.0	Lys	0.61
Sorghum	5.00	Met	0.40
Conch meal	5.00	Ca	1.10
NaCl	0.50	AP	0.54
Limestone	1.50		
Premix^a^	1.00		
Total	100.00		

a *The premix provided the following per kg of diets: VA6 000.00 IU, VD_3_1 200.00 IU, VE34.00 IU, VB_1_3.2 mg, VB_2_7.2 mg, VB_12_ 0.1 mg, biotin1.34 mg, folic acid 1.34 mg, nicotinic acid 15.00 mg, pantothenic acid 7.50 mg, Cu (as copper sulfate) 12.00 mg, Fe (as ferrous sulfate) 35.00 mg, Zn (as zinc sulfate) 35.00 mg, Mn (as manganese sulfate) 55.00 mg, I (as potassium iodide) 0.25 mg*.

b *Nutrient levels was a calculated using NRC (1994) values*.

### Measurement Index and Method

#### Sample Collection

Body weight (BW) was measured on days 0 and 28 before feeding, and the average daily gain (ADG) was calculated. At the completion of the feeding trial, to determine meat quality, four squabs with identical growth, with good health and nutrition were randomly selected for slaughter. Within 2 h after slaughter, pectoral meat samples were taken from each squab according to the technical specifications of the NY/T823-2004 squab muscle quality determination, and samples were stored in marked self-sealed bags and were then vacuum-packed. The content of inosinic acid in pectoral of squabs was determined by high-performance liquid chromatography (22). The meat samples were pretreated according to the GB/T 17376-2008. The fatty acid content was determined according to Chen et al. (22).

One squab was randomly selected from each replicate, and duodenum, jejunum and ileum were ligated after humanely sacrificing and dissecting. Each intestinal segment were collected into EP tubes and fixed in 4% paraformaldehyde. After fixation for 24 h, the sections were stored in 70% ethanol and embedded in paraffin (EG1150h, LEIC, Germany). The tissue sections were then embedded (rotating microbodies, RM2225, LEIC, Germany), stained with hematoxylin and eosin (23), and the villus height (VH) and crypt depth (CD) were measured under a light microscope (S4E, LEIC, Germany) and Image analyzer (Image-Proplus 5.0). Ten intestinal tissue regions with intact villi were selected and photographed under the microscope's 50-fold field of vision. Image-Proplus 5.0 image analysis software was used to measure the VH and CD of individual sample.

The contents of ileum were collected from each squab. then quickly frozen in liquid nitrogen, sent to the laboratory and stored at −80 °C for further analysis of microbiota.

#### Genomic DNA Extraction and PCR Amplification

The DNA was extracted from the intestinal contents using the QIAamp rapid DNA template mini-kit (Qiagen, Hilden, Germany), and the DNA concentration and purity were monitored on a 0.8% agarose gel, which was diluted to 1 ng/μL with sterile water according to the concentration.

All PCR reactions were performed using DNA 1 μL, F-primer/R-primer (20 μmol/L) 0.4 μL, 2 × Phusion® High-Fidelity PCR Master Mix with GC Buffer (New England Biolabs [Beijing] Ltd., China) 10 μL and Nucleotide-free water 8.2 μL, with thermal cycling conditions consisting of initial denaturation at 94°C for 3 min, followed by 35 cycles of 94°C for 45 s, 50°C for 60 s, and 72°C for 90 s, with a final extension step at 72°C for 10 min. Following separation using 2% agarose gel electrophoresis (in TAE buffer), PCR products in the bright main strip between 400 and 450 bp were mixed in equidensity ratios and purified with a Qiagen Gel Extraction Kit (QIAGEN, Dusseldorf, Germany).

#### Preparation of Sequencing Library and High-Throughput Sequencing

The library was constructed according to the TruSeq® Nano DNA LT Library Prep Kit (Illumina, San Diego, USA), and the V3–V4 regions of 16S rDNA were amplified with specific primers (338F/806R):

338F: 5′-ACT CCT ACG GG AGG CAG CAG-3′

806R: 5′-GGA CTA CHV GGG TWT CTA AT-3′

Based on the manufacturer's recommendations, the TruSeq® DNA PCR-sample-free preparation kit (Illumina, San Diego, USA) was used to generate the sequencing library, and the barcode was added. The Illumina MiSeq platform was used for sequencing, and 250 bp paired end readings were generated. Library construction and sequencing were performed by Shanghai Partheno Biotech Co. Ltd.

### Data Analysis

#### Processing of Original Double-Ended Sequencing Data

Original sequencing was performed using Cut-adapt shear low-quality reads (V1.9.1, https://readthedocs.org/projects/cutadapt/). The barcode was then removed with the primer sequences, and preliminary quality control was conducted to obtain the original data (raw reads). The original data sequence (UCHIME Algorithm, http://www.drive5.com/usearch/manual/uchime_algo.html) (24) was compared with the species annotation database to detect chimera sequences, which were then removed to obtain the final valid data (clean reads).

#### Classification of Operational Taxonomic Units

Operational taxonomic units (OTU) or amplicon sequence variants (ASV) clustering was performed based on 97% similarity using Uparse v7.0.1001 (https://drive5.com/uparse/). The Mothur method was used with SILVA DE (http://www.arb-silva./) (25) and the Greengenes database (Release 13.8, https://greengenes.secondgenome.com/) (26) of the annotated OTUs were used to represent the species sequence analysis, with a threshold of 70% used to ensure the accuracy of the results of the analysis. The OTUs whose abundances were <0.001% of the total sequencing volume of the whole sample were removed (27). The OTU abundance data were normalized using the sequence number standard corresponding to the sample with the lowest sequence. The OTU of each sample was identified at the species level based on the normalized data output and the microbial alpha and beta diversity analysis.

#### Alpha Diversity Analysis

Chao1 and the observed species index were used to represent richness, and the Shannon Index was used to represent diversity. In this experiment, the Chao1, Observed Species Index, and the Shannon Index were mainly used to reflect species differences among the treatments. The indexes were calculated using QIIME (Version 1.9.1) and displayed using R software (Version 2.15.3).

#### Microbial Community Structure at the Phylum and Genus Levels

Stacked histograms of species composition, are the most commonly used means of representing the composition of diverse species. By statistically analyzing the feature table after the removal of singletons, the composition distribution of each sample at six classification levels (phylum, class, order, family, genus, and species) was visualized.

#### Beta Diversity Analysis

The beta diversity analysis was used to evaluate the differences in species complexity between samples, and QIIME software (Version 1.9.1) was used to calculate the Unifrac distance. A multivariate analysis of variance based on the substitution test (which assumes that the samples within groups are more similar than the samples between groups) was also used, along with the Adonis function of the Vegan package in R language, which is usually used to show intra-group and component differences. The default random forests (28) algorithm was selected for the analysis.

#### MetagenomeSeq Analysis

The MetagenomeSeq analysis was used to find the ASV or OTU with statistically significant differences among sample groups and whether these ASV or OTU differences have a tendency of enrichment at different classification levels. The MetagenomeSeq method was used to compare the sample groups in pairs (the default). Its results were further presented through the Manhattan plot to show the difference in the ASV or OTU and the taxonomic annotation. Compared to other methods, it not only shows the whole data, but can also quickly find the target ASV or OTU and determine the specific classification location and significance of the target.

#### Data Statistics and Processing

The data were collected using Microsoft Excel 2017, one-way ANOVA in SPSS 24.0 was used to analyze the dissimilarity among treatments, where after Duncan multiple comparative test was proceeded for the data with significant imparity. Data were expressed as mean ± SD. Values of *P* < 0.05 were considered statistically remarkable. The intestinal microbiota diversity analysis was carried out on the Genes Cloud Platform (https://www.genescloud.cn).

## Results

### Growth Performance and Meat Quality

As shown in Table 2, initial BW and meat quality were not significantly affected by the dietary treatments (*P* > 0.05). The average daily gain and final BW increased significantly in the groups CSB-275 and CCP-110 in comparison with the control group (*P* < 0.05).

**Table 2:** Effects of coated sodium butyrate (CSB) and polysaccharides from *Cordyceps cicadae* (CCP) on growth performance and meat quality of squabs.

**Items**	**Groups**						
	**Control**	**CSB-275**	**CSB-550**	**CSB-1100**	**CCP-27.5**	**CCP-55**	**CCP-110**
Initial BW g	15.85 ± 0.48	15.45 ± 0.44	15.85 ± 0.50	15.55 ± 0.11	15.88 ± 0.80	15.55 ± 0.22	15.48 ± 0.19
Final BW g	478.13 ± 27.11b	535.73 ± 20.57a	490.80 ± 28.15ab	496.80 ± 37.07ab	481.23 ± 19.33ab	503.73 ± 28.45ab	537.68 ± 20.59a
ADG	16.51 ± 0.97^b^	18.58 ± 0.72^a^	16.96 ± 1.02^ab^	17.19 ± 1.32^ab^	16.62 ± 0.72^ab^	17.43 ± 1.01^ab^	18.65 ± 0.74^a^
Inosinic acid mg/kg	1630.73 ± 119.24	1672.83 ± 163.22	1529.73 ± 129.58	1432.35 ± 123.37	1580.32 ± 124.86	1449.11 ± 78.60	1670.63 ± 162.89
Fatty acid %	3.00 ± 0.30	3.02 ± 0.18	2.65 ± 0.40	2.93 ± 0.47	3.68 ± 0.89	3.01 ± 0.37	3.03 ± 0.08

*In the same row, values with no letter or the same small letter superscripts mean no significant difference (P > 0.05), while with different small letter superscripts mean significant difference (P < 0.05)*.

*CSB, coated sodium butyrate; CCP, polysaccharides from Cordyceps cicadae; BW, Body weight, ADG, average daily gain*.

### Intestinal Morphology

As demonstrated in Table 3, Supplementary Figure 1, the duodenal VH in squabs in the experimental groups other than groups CSB-1100 and CCP-110 increased (*P* > 0.05); the duodenal CD in squabs in the experimental groups decreased (*P* > 0.05); and the VH/CD of groups CSB-275, CSB-550, and CCP-55 were higher than those of the control group (*P* < 0.05). The VH of the jejunum in the experimental groups were higher than those of the control group (*P* < 0.05); the CD of CSB-1100 was lower than that of the control group (*P* < 0.05); and the VH/CD of CSB-550, CSB-1100, CCP-55 and CCP-1000 was higher than that of control (*P* < 0.05) (Table 3, Supplementary Figure 2). The VH of the ilea of squabs in the CSB-275, CSB-550, and CCP-110 groups were higher than those of the control group (*P* < 0.05); there was no difference in CD of ileum among groups (*P* > 0.05); the VH/CD of CCP-110 was higher than that of the control group (*P* < 0.05) (Table 3, Supplementary Figure 3).

**Table 3:** Effects of coated sodium butyrate (CSB) and polysaccharides from *Cordyceps cicadae* (CCP) on the villi height (μm), Crypt depth (μm) and VH/CD of the duodenum, jejunum, and ileum of squabs.

**Items**	**Groups**						
	**Control**	**CSB-275**	**CSB-550**	**CSB-1100**	**CCP-27.5**	**CCP-55**	**CCP-110**
**Duodenum**							
Villi height	988.18 ± 72.86	1059.97 ± 55.41	1006.53 ± 76.52	941.58 ± 58.32	1055.99 ± 79.07	1139.23 ± 57.03	97.97 ± 89.52
Crypt depth	155.72 ± 19.08	136.00 ± 10.91	134.66 ± 9.72	146.03 ± 12.25	147.66 ± 23.58	151.58 ± 14.29	139.79 ± 9.48
VH/CD	6.44 ± 0.93^b^	7.84 ± 0.72^a^	7.51 ± 0.79^a^	6.48 ± 0.58^ab^	7.30 ± 0.98^ab^	7.60 ± 0.91^a^	6.74 ± 0.73^ab^
**Jejunum**							
Villi height	264.94 ± 55.63^b^	380.54 ± 45.31^a^	335.85 ± 91.72^a^	367.43 ± 90.38^a^	313.45 ± 90.52^a^	332.20 ± 64.98^a^	417.87 ± 78.74^a^
Crypt depth	108.67 ± 14.33^a^	105.30 ± 14.17^ab^	89.09 ± 21.37^ab^	70.80 ± 7.66^b^	86.15 ± 21.68^ab^	75.87 ± 18.94^ab^	97.08 ± 27.53^ab^
VH/CD	2.44 ± 0.47^b^	3.64 ± 0.35^ab^	3.79 ± 0.73^a^	5.14 ± 0.91^a^	3.67 ± 0.94^ab^	4.45 ± 0.53^a^	4.50 ± 0.84^a^
**Ileum**							
Villi height	148.15 ± 36.96^b^	261.65 ± 45.43^a^	240.03 ± 62.57^a^	228.09 ± 43.08^ab^	215.42 ± 63.46^ab^	205.88 ± 16.17^ab^	295.92 ± 44.18^a^
Crypt depth	71.29 ± 6.74	70.34 ± 7.39	79.55 ± 10.01	68.60 ± 5.08	84.46 ± 8.58	79.60 ± 9.86	64.12 ± 10.00
VH/CD	2.08 ± 0.45^b^	3.77 ± 0.84^ab^	2.99 ± 0.58^ab^	3.37 ± 0.78^ab^	2.53 ± 0.57^ab^	2.64 ± 0.48^ab^	4.68 ± 0.80^a^

*In the same row, values with no letter or the same small letter superscripts mean no significant difference (P > 0.05), while with different small letter superscripts mean significant difference (P < 0.05)*.

*CSB, coated sodium butyrate; CCP, polysaccharides from Cordyceps cicadae; VH/CD, villi height/crypt depth*.

### Bacterial Analyses of Ileal Contents

#### Data Summary

**Figure 1:**
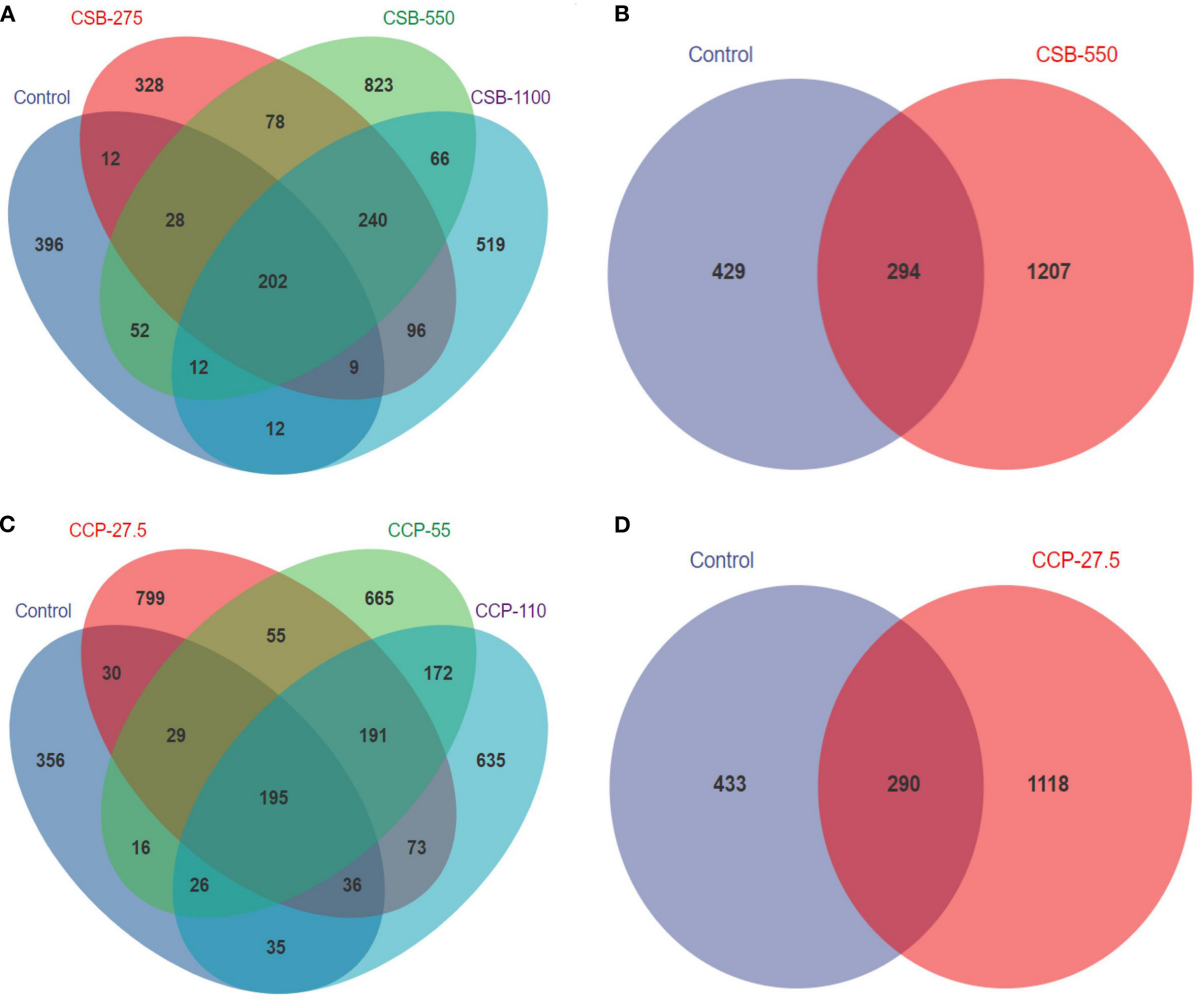
The number of shared and specific operational taxonomic units (OTUs) in each group. Each circle represents one group; the number in the overlapping portion of the circles represents the number of shared OTUs between the different groups, and the number in the non-overlapping portion of the circles represents the OTUs specific to each group. **(A)** Between squabs fed the CSB diet and the control diet. **(B)** Between groups CSB-550 and control. **(C)** Between squabs fed the CCP diet and the control diet. **(D)** Between groups CCP-27.5 and control.

After the mass fractions of the sequence readings were determined, sequencing errors removed, and chimeric filtering performed, we identified 3,102,585 sequences. A total of 6,864 OTUs were obtained and successfully classified at the domain level using a classifier. Making Venn diagram using OTU abundance table. There were 202 and 195 OTUs in common between the experimental groups fed CSB and CCP and the control group, respectively (Figure 1). CSB-550 and CCP-27.5 had the highest numbers of unique OTUs. The number of OTUs in the experimental groups was higher than in the control group, indicating that CSB and CCP changed with the species composition and abundance of the ileal microflora in squabs.

#### Alpha Diversity Analysis

**Figure 2:**
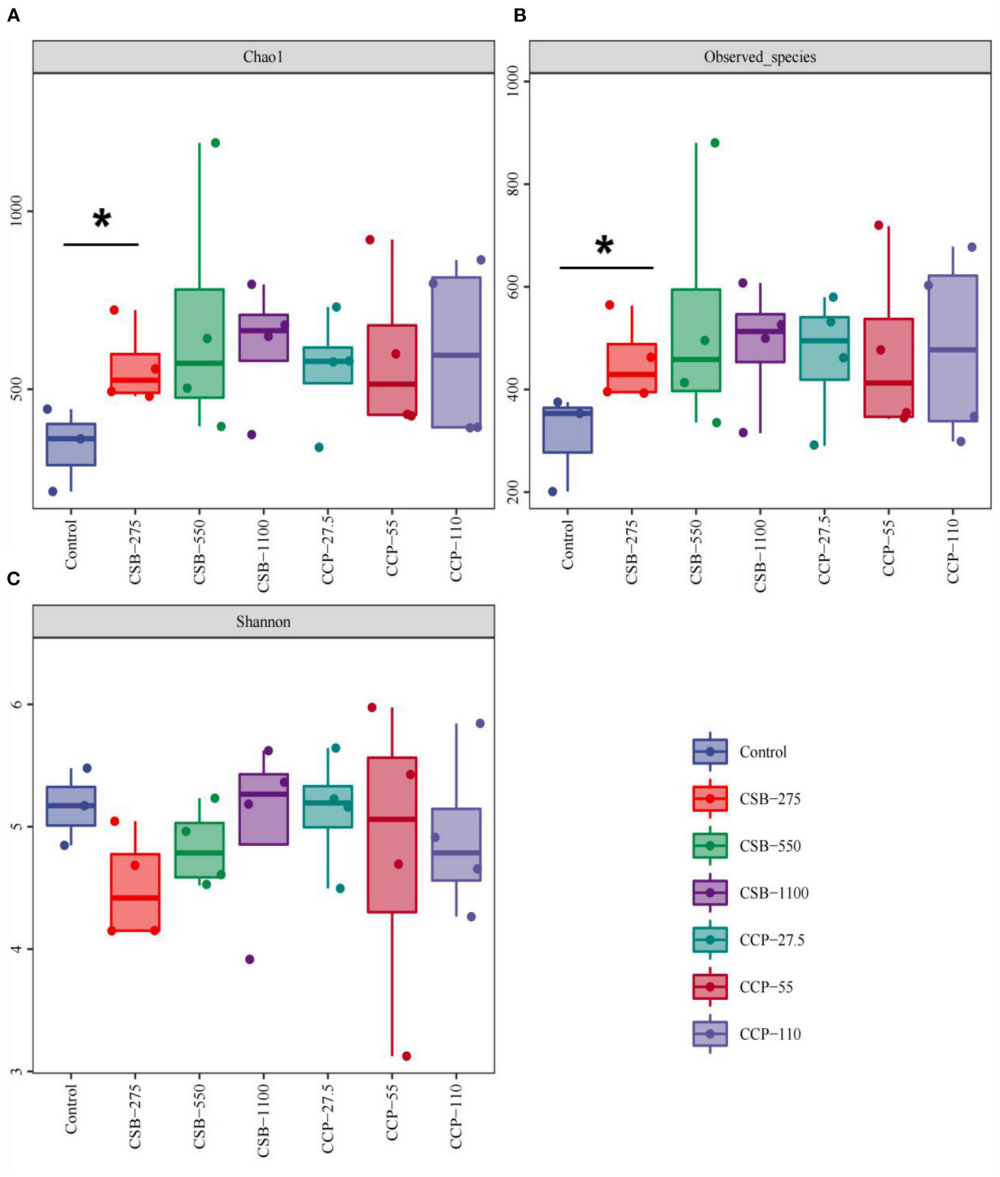
Richness measures (alpha diversity) for the ileal bacterial communities in different concentrations of CSB and CCP. Asterisks denote significant differences between groups (*P* < 0.05). **(A)** Chao 1 indices. **(B)** Observed species. **(C)** Shannon indices.

The α diversity assessed by the Chao1, Shannon, and observed species indexes is shown in Figure 2A. The Chao1 index was used to estimate the total number of species in the measured sample. The Chao1 index of the CSB-275 group was higher than in the control group (*P* < 0.05) (Figure 2A), indicating that the addition of CSB increased the richness of ileal microflora. Moreover, the higher the Shannon index, the higher the community diversity in the tested samples. As shown in Figure 2B, there was no difference in the Shannon index between the experimental groups and the control group (*P* > 0.05). The observed species index refers to the total number of species observed in the sequence of the sample. As shown in Figure 2C, the observed species index of the group CSB-275 was higher than that of the control group (*P* < 0.05), which was consistent with the Chao1 index, and there was no difference between the control and the other groups (*P* > 0.05).

#### Microbial Community Structure at the Phylum and Genus Levels

**Figure 3:**
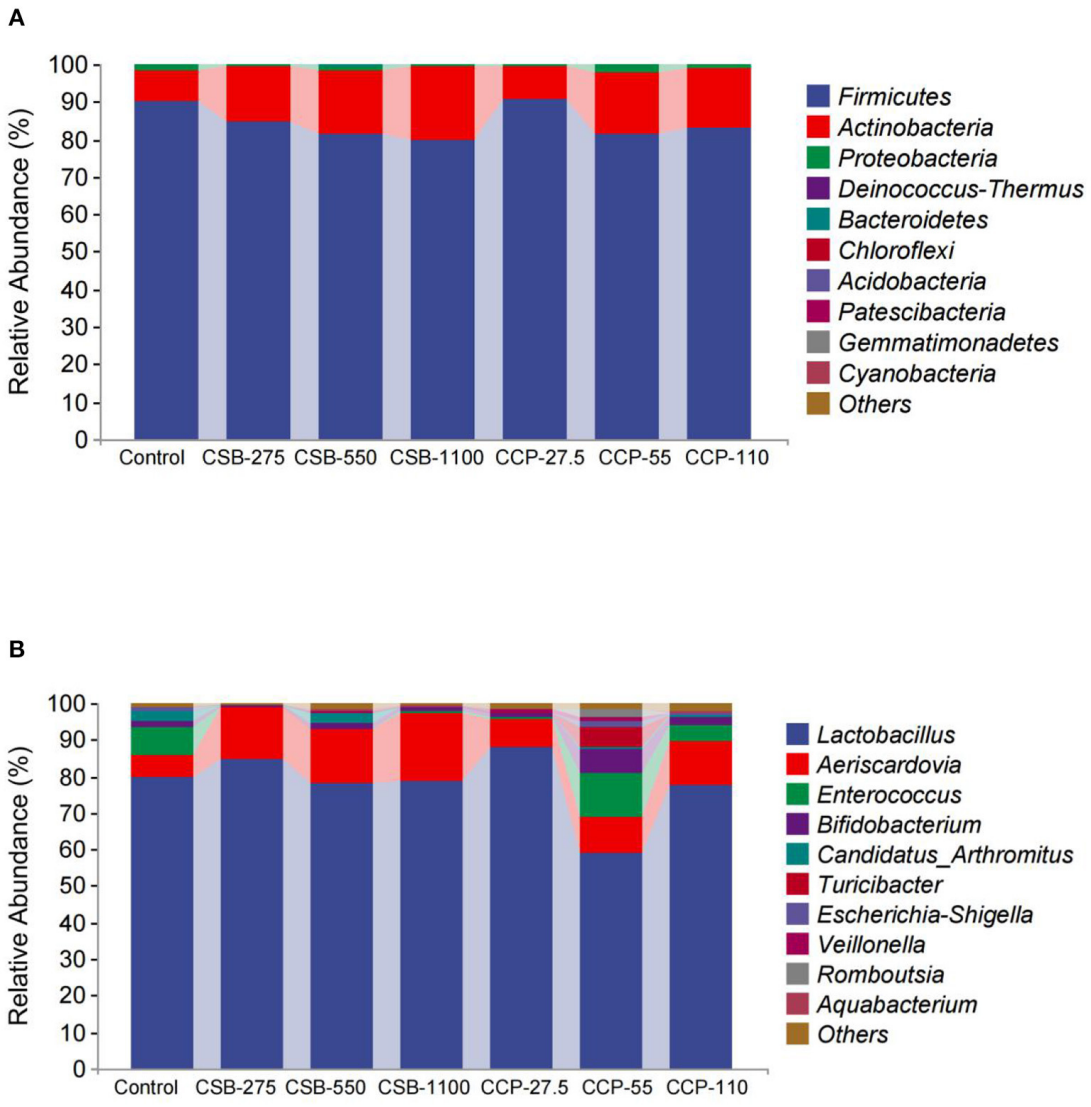
Taxonomic classification of each group at the phylum and genus level: **(A)** phylum level; **(B)** genus level.

In this experiment, 10 different phyla were identified in the squab ileal microflora (Table 4, Figure 3A). At the phylum level, whole content samples of the test and control groups had nearly identical community structures. In the control group, *Firmicutes* were the most abundant bacteria (90.05%), followed by *Actinobacteria* (8.35%), *Proteobacteria* (1.5%), and *Deinococcus–Thermus* (0.06%). The abundances of *Actinobacteria* in the CSB-550, CSB-1100, and CCP-55 groups were higher than in the controls (*P* < 0.05), but there were no differences in the abundances of the other bacteria (*P* > 0.05).

**Table 4:** Abundance of main microbiota (accounting for ≥0.05% of the total sequences in at least one of the samples) in each group (abundance of the phyla is expressed as a percentage).

**Items**	**Groups**						
	**Control**	**CSB-275**	**CSB-550**	**CSB-1100**	**CCP-27.5**	**CCP-55**	**CCP-110**
*Firmicutes*	90.05 ± 4.07	84.87 ± 6.18	81.46 ± 7.31	79.89 ± 6.95	90.47 ± 5.78	81.35 ± 8.37	82.88 ± 8.21
*Actinobacteria*	8.35 ± 3.86^b^	14.79 ± 4.18^ab^	16.69 ± 5.3^a^	19.59 ± 3.88^a^	9.01 ± 2.88^ab^	16.61 ± 3.79^a^	16.08 ± 8.24^ab^
*Proteobacteria*	1.50 ± 0.13	0.30 ± 0.01	1.53 ± 0.06	0.46 ± 0.01	0.43 ± 0.14	1.81 ± 0.13	0.94 ± 0.14
*Deinococcus-Thermus*	0.02 ± 0.02	0.06 ± 0.01	0.03 ± 0.01	0.05 ± 0.01	0.03 ± 0.01	0.14 ± 0.08	0.08 ± 0.01

*In the same row, values with no letter or the same small letter superscripts mean no significant difference (P > 0.05), while with different small letter superscripts mean significant difference (P < 0.05)*.

*CSB, coated sodium butyrate; CCP, polysaccharides from Cordyceps cicadae*.

At the genus level (Table 5, Figure 3B), *Lactobacillus, Aeriscardovia, Enterococcus*, and *Bifidobacterium* were the four main genera identified; *Lactobacillus* made up the largest proportion, and the proportions decreased in the order listed above. The abundance of *Lactobacillus* in the CCP-55 squab ileal microflora was lower than in the other groups (*P* < 0.05). The abundances of *Aeriscardovia* were higher in the CSB-275, CSB-550, CSB-1100, and CCP-110 groups compared with the control group (*P* < 0.05), while the abundances of *Enterococci* in the CSB-275, CSB-550, CSB-1100, and CCP-27.5 groups were lower than in the control group (*P* < 0.05). The relative abundance of *Bifidobacterium* in the CCP-55 group was higher than in the control group (*P* < 0.05).

**Table 5:** Abundance of main microbiota (accounting for ≥0.05% of the total sequences in at least one of the samples) in each group (abundance of the genera is expressed as a percentage).

**Items**	**Groups**						
	**Control**	**CSB-275**	**CSB-550**	**CSB-1100**	**CCP-27.5**	**CCP-55**	**CCP-110**
*Lactobacillus*	79.50 ± 9.22^a^	84.71 ± 6.23^a^	78.12 ± 9.22^a^	78.88 ± 7.33^a^	88.21 ± 5.43^a^	56.42 ± 8.15^b^	77.53 ± 8.89^a^
*Aeriscardovia*	6.27 ± 1.47^b^	14.50 ± 3.86^a^	14.73 ± 6.05^a^	18.44 ± 6.75^a^	7.48 ± 0.61^ab^	9.91 ± 1.09^ab^	12.19 ± 3.21^a^
*Enterococcus*	7.11 ± 1.12^ab^	0.07 ± 0.02^c^	0.13 ± 0.04^c^	0.60 ± 0.26^c^	0.63 ± 0.17^c^	11.93 ± 1.94^a^	4.78 ± 0.74^bc^
*Bifidobacterium*	1.96 ± 0.16^ab^	0.63 ± 0.27^b^	1.60 ± 0.20^b^	0.96 ± 0.19^b^	0.68 ± 0.16^b^	6.43 ± 0.37^a^	2.400.60^ab^

*In the same row, values with no letter or the same small letter superscripts mean no significant difference (P > 0.05), while with different small letter superscripts mean significant difference (P < 0.05)*.

*CSB, coated sodium butyrate; CCP, polysaccharides from Cordyceps cicadae*.

#### Beta Diversity Analysis

**Figure 4:**
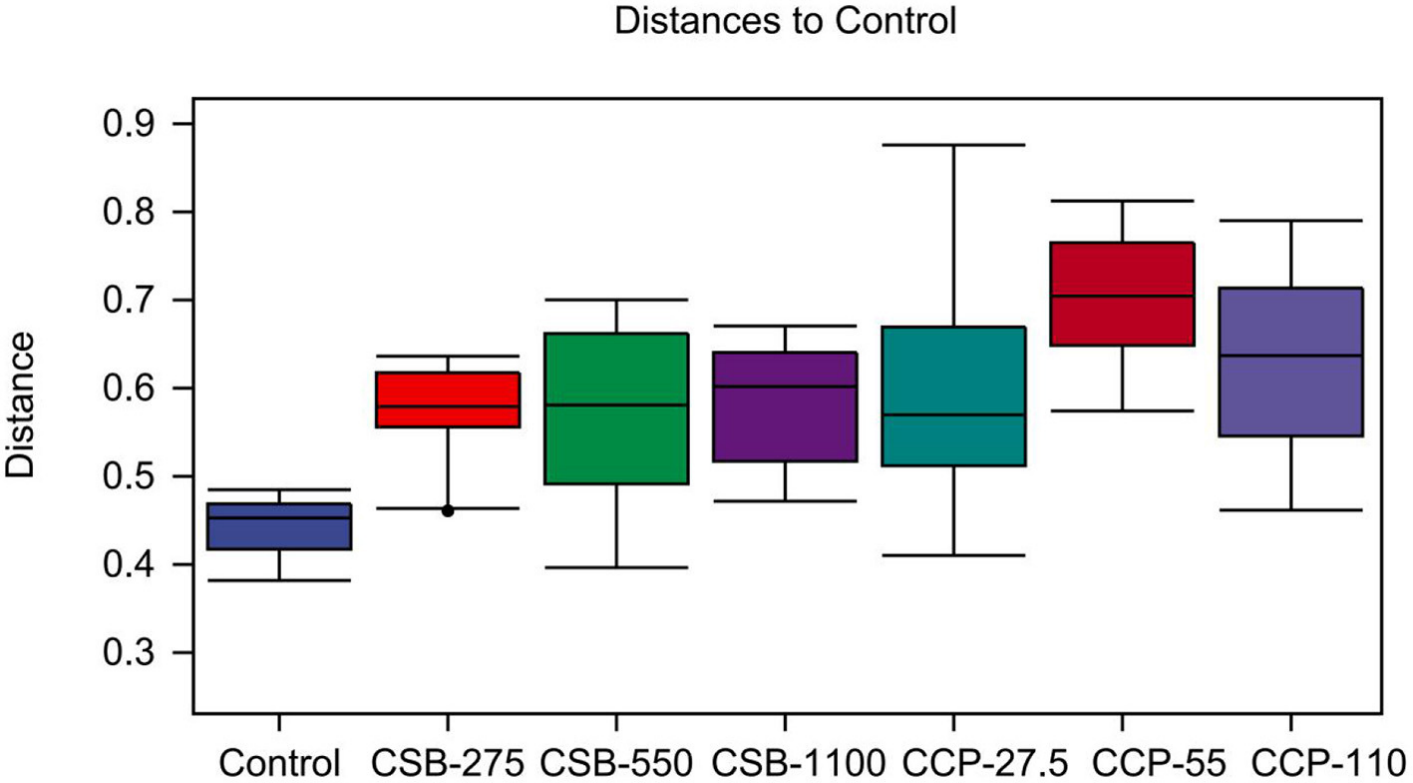
Unweighted UniFrac distances for within and between study comparisons. It mainly reflects the analysis of differences between groups.

This quartile was used to calculate the distances between different groups, and the differences in the distance distribution within and between groups were compared (Figure 4). The difference in the sample distribution in the control was lower than that in the CSB-275 group (*P* < 0.05), but no differences were found in other groups (*P* > 0.05).

**Figure 5:**
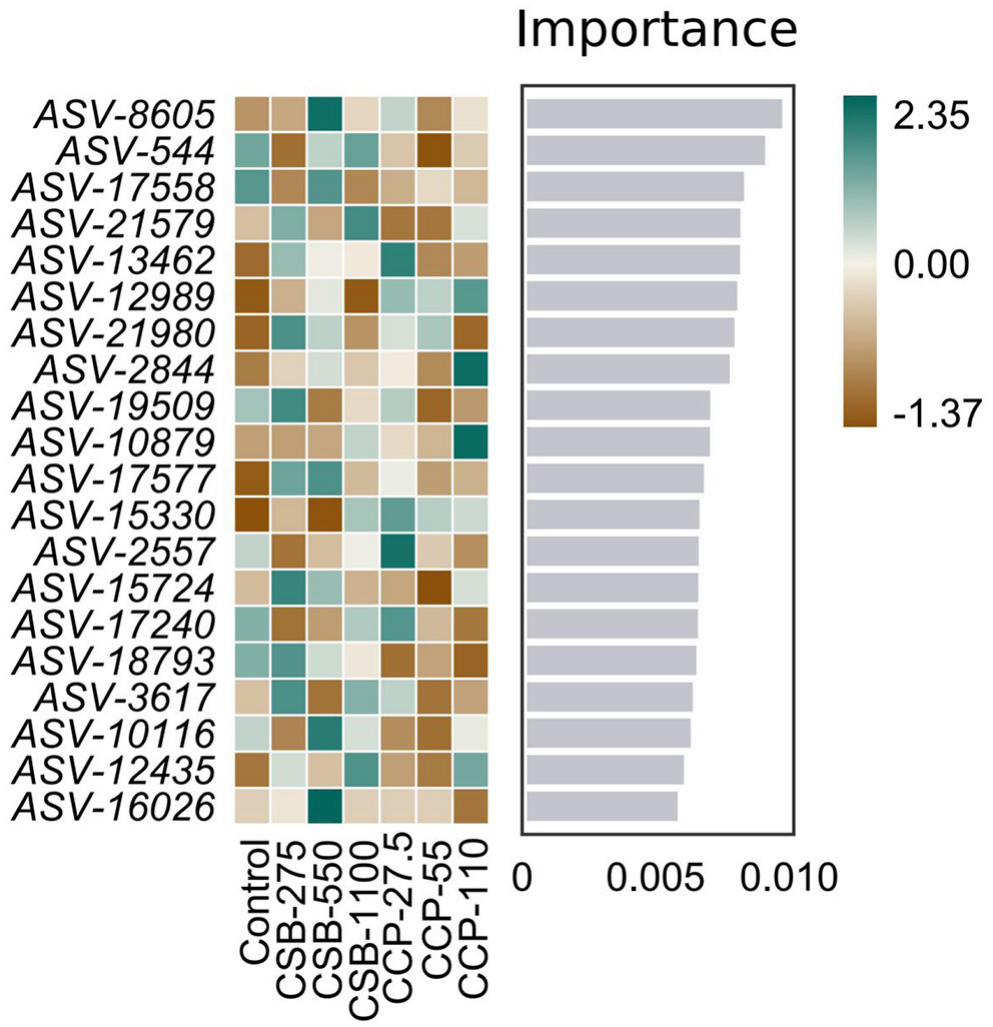
Random forest distribution results. The darker the color, the higher or lower the flora abundance, so as to reflect the flora abundance difference of each ASV.

Because the random forest distribution map is a non-linear classifier, the complex non-linear interdependence among the variables can be mined, and the key ASV that distinguishes differences between two groups of samples can be identified. Figure 5 shows the abundance distribution of the species in each group; from top to bottom, the importance of species to the model decreases, with species at the top indicating differences between the groups.

#### MetagenomeSeq Results Interpretation

**Figure 6:**
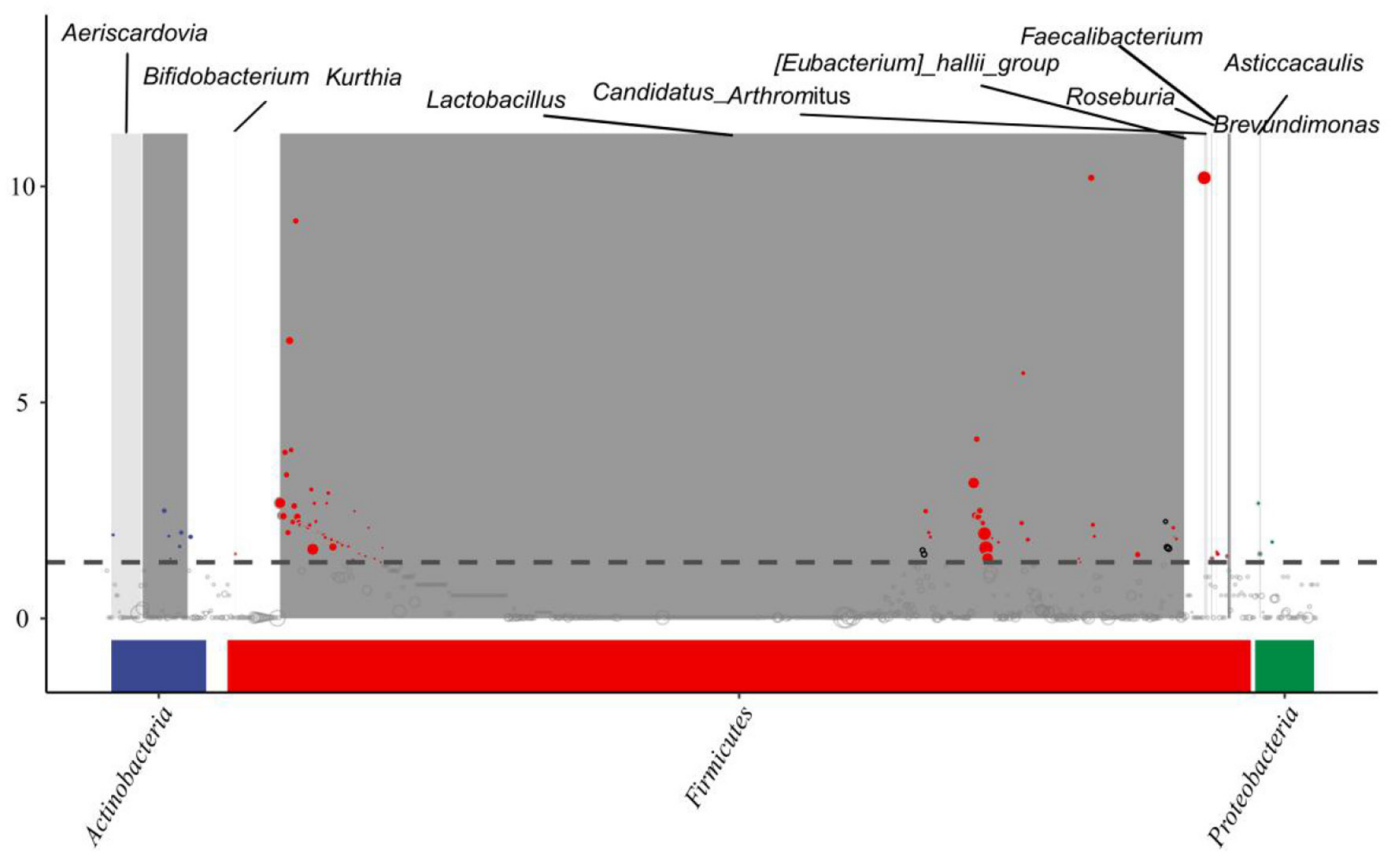
MetagenomeSeq interpretation of the results. Significant differences were marked by colored dots or rings, non-significant differences were represented by gray rings.

As shown in Figure 6, we tried to identify the ASV by differences among the sample groups and then determine whether these differences tend to be enriched at different classification levels. Here, the metagenomeSeq method was used to compare pairs of sample groups. We further analyzed the data using a Manhattan plot to display the metagenomeSeq analysis results to determine if there was a significant difference in microbial species between two sample groups; a *P*-value < 0.05 used as the threshold to indicate significant differences. Differences were mainly found in the *Firmicutes, Actinobacteria*, and *Proteobacteria*.

## Discussion

SB plays a significant role in promoting the growth of animals, but its wide application is limited because of its special properties and odor (29). However, CSB can mask this odor, thus promoting feeding in animals can improve the immune system of animals (30), while being a green and pollution-free animal-feed additive (31). The beneficial effects of supplementary butyrate feeding on poultry are documented and characterized by growth performance improvement and potential positive modulation of GI tract microbiota (6, 32). CCP possess significant antioxidant and anti-aging activities (33), it could enhance the immune capacity of the body (14, 15). In the present study, the pigeons fed the 275 mg/kg CSB and 110 mg/kg CCP diet showed an increase in final BW and ADG efficiency in comparison with the CON group, which is consistent with the findings of a previous study (34). Although studies have demonstrated the regulation of butyrate on fat accumulation (35, 36) the difference associating with different concentrations was not clearly defined. In the present study, because of large individual variation, there was no difference in meat quality.

Intestinal morphology is an important factor affecting intestinal health, especially the VH, CD, and VH/CD, and many villus projections are formed by large numbers of annular folds on the inner surface of the small intestinal wall (37), CSB can improve the absorptive capacity of the digestive tract of chicks (6, 38). Czerwiński et al. (39) found that adding 300 mg/kg of CSB to the diets of Ross 308 broilers significantly increased the VH of their jejuna. In this experiment, compared with the control group, the CSB-275, CSB-550, and CCP-55 groups had significant increases in the squab duodenal VH/CD. The inclusion of CSB and CCP in the diet can improve the whole duodenal VH/CD value of the squabs, thus improving whole duodenal absorption ability of the pigeon; of all the groups, CSB-275 and CCP-55 worked best. Compared with the control group, CSB-1100 increased the VH, CD, and VH/CD values in the jejuna of the squabs. The results indicated that CSB and CCP may affect the intestinal development of squabs. Similarly, the CSB-275, CSB-550, and CCP-55 groups had significantly increased ileal VH compared with the control group; CSB-275 and CCP-55 had the best effect, indicating that there is an optimal concentration of either CSB or CCP for improving the intestinal tissue morphology of the squabs.

Studies have shown that the diversity of flora in the digestive tracts of animals can reflect their digestive and absorption capacities because the intestinal flora play key roles in maintaining intestinal function (40). Birds without cecums, such as passerines, do not have permanent intestinal flora (41). Studies have shown that SB can significantly reduce the colonization of *Salmonella* in the intestinal tract of broilers (42). Zou et al. (32) found that SB had no effect on the α diversity of the intestinal microflora of broilers but changed their compositions and played important roles in inducing anti-inflammatory effects and regulating the microbial community. Studies have shown that polysaccharides can act as prebiotics to prevent changes in the intestinal flora (43). For example, CCP can block the TLR4/NF-κB and TGF-β1/Smad signaling pathways to prevent intestinal flora dysregulation (44). This experiment was based on high-throughput sequencing of 16S rDNA to analyze the whole ileum contents in terms of the structure and composition of floral diversity. A total of 6,864 OTUs were found in the squab intestines; 202 and 195 OTUs were shared between the CSB and CCP groups and the control group, respectively. The maximum number of OTUs were found when CSB-550 and CCP-27.5 were added, demonstrating that different additives can cause differences in squab ileal OTUs.

Higher abundance and diversity of intestinal microorganisms results in better intestinal tract health, better maintenance of the dynamic balance of the microecosystem, and insurance of normal physiological function (45). In this study, the Chao1 and the observed species indexes of the ileal microflora in the CSB-275 group were significantly higher than those of the control group, but the Shannon index was not significantly different, indicating that the addition of 275 mg/kg CSB could significantly improve the richness of squab ileal microflora but has little effect on evenness. As a dynamic ecosystem, the gut microbiome is susceptible to many environmental factors, such as dietary habits, lifestyle, age, and host genotype; diet is one of the most important determinants of the gut microbiome (46). In addition, there are differences in animal hormone levels and dietary intestinal transport time, which may play important roles in shaping the intestinal microbiome (47, 48).

Best et al. (49) reported that the dominant phyla in the intestinal contents of healthy ducks were *Bacteroidetes, Firmicutes, Proteobacteria*, and *Fusobacteria. Firmicutes* is also the dominant phylum in all vertebrates (50); this was also observed in the intestinal contents from all experimental and control group squabs in this study. *Firmicutes* are positively correlated with the ability to collect energy and absorb nutrients from feed ingredients in mice and humans (51). The relative abundance values of the main phyla reported by different groups were significantly different (52, 53). In particular, with a decrease in the level of bacterial classification, the relative abundance of bacterial genera and species reported in different studies are less similar (54). In this experiment, there was no significant differences in the abundances of *Firmicutes* at the phylum level between the experimental groups and control group, but the abundances of *Actinobacteria* in the CSB-550, CSB-1100, and CCP-55 groups were significantly higher than in the control group. Most *Actinobacteria* are beneficial and some produce substances similar to antibiotics (55). These results indicate that dietary supplementation with CSB and CCP could be beneficial to the balance of the intestinal flora of squabs and improve their intestinal health.

In this study, the intestinal flora was dominated by *Lactobacillus* at the genus level. A beneficial type of bacteria, *Lactobacillus* can improve the intestinal flora to augment the intestinal barrier effect (56), and *Lactobacillus* have been related to the biosynthesis of folic acid in the human body (57). Folic acid plays an important role in RNA and DNA biosynthesis and repair, and abundant *Lactobacillus* in meat pigeons may contribute to reproductive nutrition and prenatal health. *Lactobacillus* ferments sugars to produce lactic acid, which helps maintain health and regulates immune functions. *Enterococcus* is a Gram-positive facultative anaerobic bacterium, and while the main component of some *Enterococcus* species can reduce adverse reactions to antibiotics through regulation of the immune system (58), once they reach a certain level, intestinal infection can occur (59). In this experiment, *Lactobacilli* in the ilea of squabs in the CSB-275 and CCP-27.5 groups were higher than in the control group, while *Enterococci* in the CSB-275, CSB-550, CSB-1100, and CCP-27.5 groups were significantly lower than in the control group.

Studies have shown that *Lactobacillus* release the enzyme bile salt hydrolase, it can reduce the activity of bile salts and thus reduce lipid absorption (60). Therefore, an increase in the number of bacterial species which secrete this enzyme will have a negative impact on lipid absorption and therefore intestinal function (61). In this experiment, the analysis of the random forest distribution and metagenomeSeq results showed that there were differences among the *Firmicutes, Actinobacteria*, and *Proteobacteria*, and ASV-8605 was significantly upregulated in the CSB-550 group, while ASV-54 was significantly downregulated in the CCP-55 group. Because the ileal microflora of squabs affect intestinal health, their mechanisms of regulation should be studied further.

## Conclusions

The additives CSB and CCP had positive effects on the VH and CD of the small intestines of squabs and increased the diversity of the intestinal flora, effectively increasing the beneficial flora, and positively affecting the intestinal morphology. Under the experimental conditions, 275 mg/kg of CSB and 55 mg/kg of CCP in the feed had the best effects. The results of this experiment will provide basic data for scientific research and management of the intestinal flora in squab production.

## Data Availability Statement

The datasets presented in this study can be found in online repositories. The name of the repository and accession number can be found at: National Genomics Data Center (https://ngdc.cncb.ac.cn); PRJCA007949.

## Ethics Statement

The experiment was approved by the Animal Care Committee of Anhui Agricultural University (no. SYDW-P20190600601).

## Author Contributions

HS and YL prepared the manuscript and collected some data. TZ, GL, ZT, WX, XZ, JW, and XC collected the samples. LL and HC were responsible for the design and direction of the experiment. All authors read and approved the final version of the manuscript.

## Funding

This work was supported by Science and Technology Cooperation Project Six aspects of agriculture, rural areas and farmers of Zhejiang Province (2019SNF017); Key research and development projects of Zhejiang Province (2016C02054-16).

## Conflict of Interest

XZ was employed by Huzhou Huajia Special Breeding Co.Ltd. The remaining authors declare that the research was conducted in the absence of any commercial or financial relationships that could be construed as a potential conflict of interest.

## Publisher's Note

All claims expressed in this article are solely those of the authors and do not necessarily represent those of their affiliated organizations, or those of the publisher, the editors and the reviewers. Any product that may be evaluated in this article, or claim that may be made by its manufacturer, is not guaranteed or endorsed by the publisher.
