# Supplementary material for: Effects of Coated Sodium Butyrate and Polysaccharides From Cordyceps cicadae on Intestinal Tissue Morphology and Ileal Microbiome of Squabs
Source: Front Vet Sci. 2022 Mar 4;9:813800. doi: 10.3389/fvets.2022.813800 (PMC8931417; doi:10.3389/fvets.2022.813800)
Supplement: Supplementary file 1 [file Table_1.DOCX]

**SUPPLEMENTARY MATERIAL**

A B C


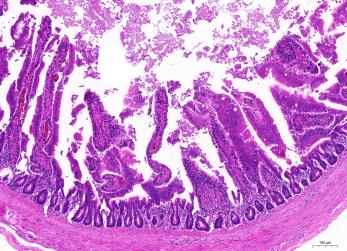

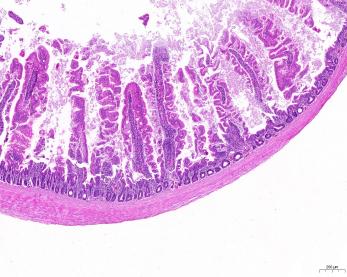

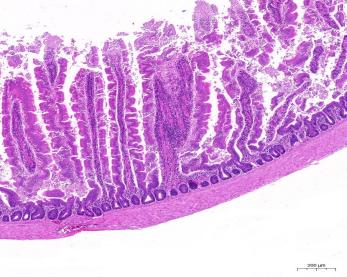


D E F


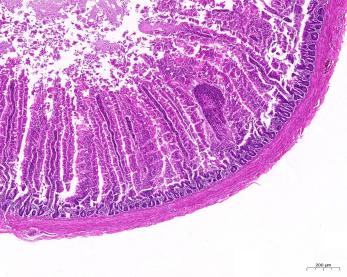

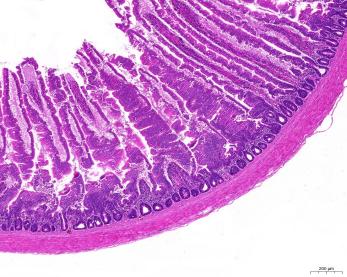

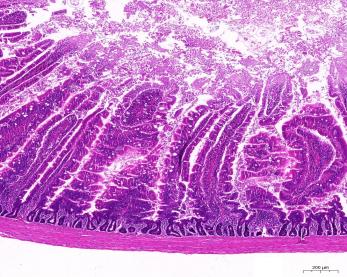


G


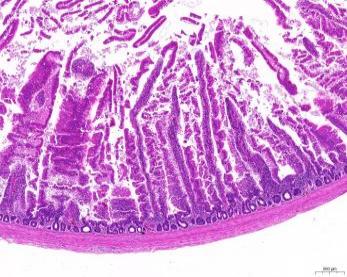


**Figure S1.** Histological section of squab duodenum. A. Control group .B. CSB-275 group. C. CSB-550 group. D. CSB-1100 group. E.CCP-27.5 group. F. CCP-110 group.

A B C


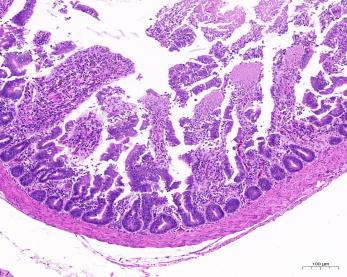

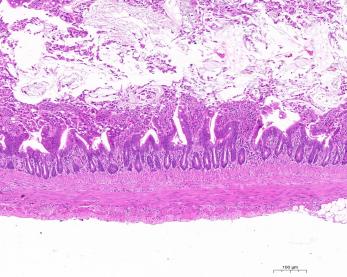

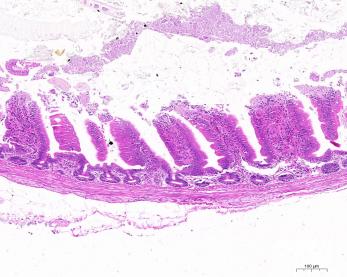


D E F


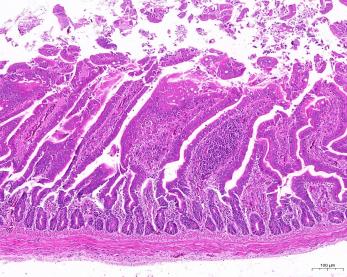

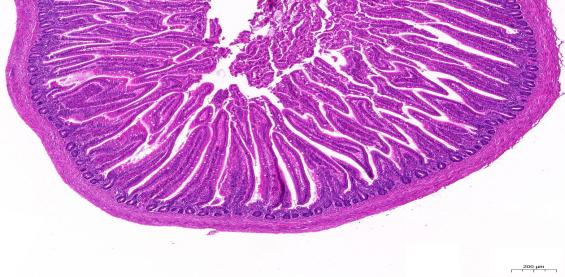

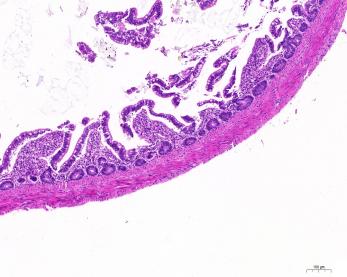


G


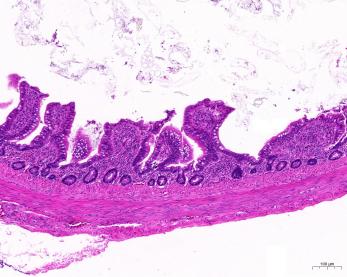


**Figure S2.** Histological section of squab jejunum. A. Control group .B. CSB-275 group. C. CSB-550 group. D. CSB-1100 group. E.CCP-27.5 group. F. CCP-110 group.

A B C


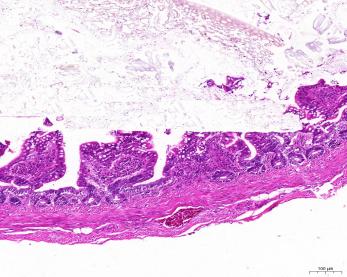

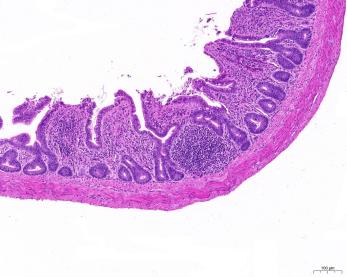

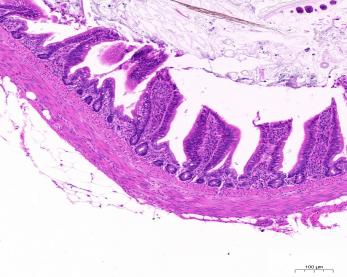


D E F


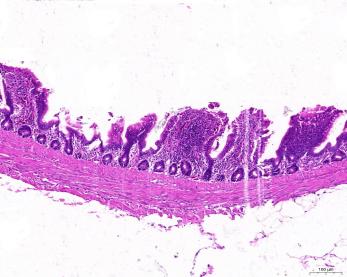

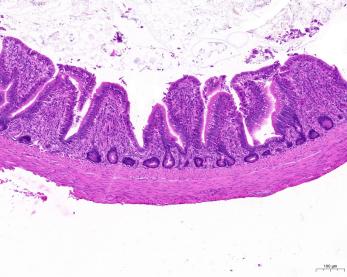

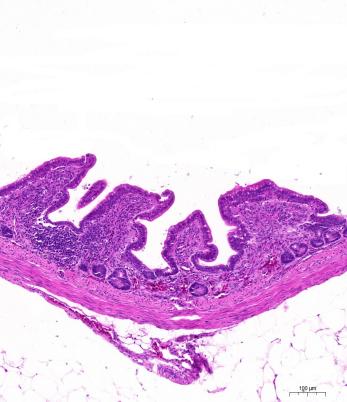


G


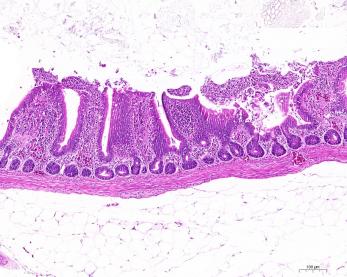


**Figure S3.** Histological section of squab ileum. A. Control group .B. CSB-275 group. C. CSB-550 group. D. CSB-1100 group. E.CCP-27.5 group. F. CCP-110 group.
